# Supplementary material for: Association between Having Cancer and Psychological Distress among Family Caregivers Using Three Years of a Nationwide Survey Data in Japan
Source: Int J Environ Res Public Health. 2021 Oct 6;18(19):10479. doi: 10.3390/ijerph181910479 (PMC8508454; doi:10.3390/ijerph181910479)
Supplement: Supplementary file 1 [file ijerph-18-10479-s001.zip › ijerph-1354892-supplementary.pdf]

Table S1. Care recipients' characteristics by family caregiver's cancer's presence

		Total	Cancer	No cancer	
		**n (%)‡**	**n (%)‡**	**n (%)‡**	**p-value †**
Sex	Male	1831 (34.8)	29 (32.2)	1802 (34.9)	0.60
	Female	3427 (65.2)	61 (67.8)	3366 (65.1)	
Age	40-64	168 (3.2)	1 (1.1)	167 (3.2)	0.20
	65-74	569 (10.8)	10 (11.1)	559 (10.8)	
	75-84	1825 (34.7)	24 (26.7)	1801 (34.9)	
	85-	2696 (51.3)	55 (61.1)	2641 (51.1)	
Primary disease for care-need	Cerebrovascular disease	1287 (24.5)	17 (18.9)	1270 (24.6)	0.47
	Heart disease	195 (3.7)	2 (2.2)	193 (3.7)	
	Cancer	107 (2.0)	4 (4.4)	103 (2.0)	
	Respiratory disease	126 (2.4)	2 (2.2)	124 (2.4)	
	Joint disease	476 (9.1)	12 (13.3)	464 (9.0)	
	Dementia	1010 (19.2)	16 (17.8)	994 (19.2)	
	Parkinson's disease	197 (3.8)	2 (2.2)	195 (3.8)	
	Diabetes	155 (3.0)	1 (1.1)	154 (3.0)	
	Visual disorder	80 (1.5)	1 (1.1)	79 (1.5)	
	Fracture	660 (12.6)	16 (17.8)	644 (12.5)	
	Spinal cord injury	112 (2.1)	3 (3.3)	109 (2.1)	
	Old age	853 (16.2)	14 (15.6)	839 (16.2)	
Certified level for care-need	Support level 1	480 (9.1)	8 (8.9)	472 (9.1)	0.84
	Support level 2	665 (12.7)	9 (10.0)	656 (12.7)	
	Care level 1	1086 (20.7)	22 (24.4)	1064 (20.6)	
	Care level 2	1236 (23.5)	23 (25.6)	1213 (23.5)	
	Care level 3	878 (16.7)	11 (12.2)	867 (16.8)	
	Care level 4	539 (10.3)	11 (12.2)	528 (10.2)	
	Care level 5	374 (7.1)	6 (6.7)	368 (7.1)	
Caring hour	Almost a day	1244 (23.7)	24 (26.7)	1220 (23.6)	0.44
	Half a day	649 (12.3)	11 (12.2)	638 (12.4)	
	2-3 hours	711 (13.5)	7 (7.8)	704 (13.6)	
	Sometimes when needed	2654 (50.5)	48 (53.3)	2606 (50.4)	

Note: † Determined by chi-square test. ‡ Percentages do not sum to 100% due to rounding. § Monthly expenditure Low/Middle: <75000 JPY per month. Monthly expenditure High: =>75000 JPY per month. (100 JPY = $1 American dollar approx.).

Table S2. Associations between family caregivers' and care-recipients' characteristics and family caregivers' distress, based on the same regression analysis as Table 2

Variable	Categories	Adjusted[†]		
		**RR**	**95% CI**	**p-value**
Family **caregivers' characteristics**				
Disease[‡]	Asthma	1.46	1.18 - 1.81	0.000
	Lower back pain	1.39	1.25 - 1.54	0.000
	Rheumatoid arthritis	1.20	0.94 - 1.52	0.141
	Angina/myocardial infarction	1.18	1.00 - 1.41	0.054
	Gastroduodenal disease	1.17	0.99 - 1.38	0.058
	Liver disease	1.17	0.94 - 1.45	0.166
	Osteoporosis	1.15	0.99 - 1.34	0.077
	Arthropathy	1.13	0.98 - 1.30	0.091
	Eye disease	1.08	0.96 - 1.22	0.211
	Fracture	1.08	0.79 - 1.46	0.628
	Diabetes	1.06	0.93 - 1.22	0.358
	Stroke	1.03	0.79 - 1.33	0.838
	Hypertension	0.93	0.85 - 1.02	0.142
Sex	Male (reference)	-		
	Female	1.22	1.09 - 1.37	<0.001
Age category	40-64 (reference)	-		
	65-74	0.85	0.75 - 0.97	0.012
	75-84	0.89	0.75 - 1.07	0.224
	85+	0.99	0.77 - 1.27	0.923
Education history	Elementary/junior high school (reference)	-		
	High school	0.96	0.87 - 1.05	0.341
	University/graduate school	0.87	0.78 - 0.98	0.023
Job status	Having any job (reference)	-		
	Not having a job	1.09	0.99 - 1.20	0.073
Monthly[§] expenditure	Less than 7.5 (x10000 yen) per month (reference)	-		
	From 7.5 (x10000 yen) per month	1.01	0.93 - 1.09	0.840
Current smoking status	Yes (reference)	-		
	No	0.88	0.78 - 0.99	0.031
Relationship with care-recipient	Spouse (reference)	-		
	Son/daughter	1.15	0.95 - 1.39	0.153
	Son-in-law/daughter-in-law	0.94	0.76 - 1.16	0.566
	Parent	1.02	0.66 - 1.59	0.929
	Other relative	0.88	0.63 - 1.21	0.426
Help from family members	Having help from family members (reference)	-		
	Not having help from family members	0.98	0.90 - 1.06	0.591
Use of formal help services	Using any formal help services (reference)	-		
	Not using any formal help services	0.87	0.81 - 0.95	0.002
**Care-recipients' characteristics**				
Sex	Male (reference)	-		
	Female	0.94	0.84 - 1.04	0.239
Age category	40-64 (reference)	-		
	65-74	0.92	0.72 - 1.17	0.502

	75-84	0.85	0.66 - 1.10	0.213
	85-	0.76	0.58 - 1.00	0.052
Primary disease for care-need	Cerebrovascular disease (reference)	-		
	Heart disease	0.93	0.75 - 1.16	0.516
	Cancer	1.07	0.84 - 1.37	0.575
	Respiratory disease	1.13	0.89 - 1.43	0.332
	Joint disease	1.07	0.92 - 1.25	0.396
	Dementia	1.05	0.93 - 1.18	0.430
	Parkinson's disease	0.93	0.76 - 1.15	0.503
	Diabetes	1.10	0.89 - 1.36	0.389
	Visual disorder	0.84	0.58 - 1.21	0.356
	Fracture	1.01	0.88 - 1.17	0.857
	Spinal cord injury	0.94	0.71 - 1.24	0.660
	Old age	0.97	0.84 - 1.12	0.709
Certified level for care-need	Support level 1 (reference)	-		
	Support level 2	0.97	0.80 - 1.16	0.714
	Care level 1	1.05	0.89 - 1.24	0.541
	Care level 2	1.05	0.89 - 1.24	0.535
	Care level 3	1.09	0.92 - 1.30	0.326
	Care level 4	1.06	0.88 - 1.28	0.512
	Care level 5	0.92	0.74 - 1.14	0.465
Caring hour	Almost a day (reference)	-		
	Half a day	0.96	0.85 - 1.08	0.468
	2-3 hours	0.88	0.77 - 1.00	0.051
	Sometimes when needed	0.74	0.66 - 0.82	<0.001

Abbreviations: RR, risk ratio. CI, confidence interval. †Poisson regression analysis adjusted by family caregivers' and care-recipients' variables. ‡Information of cancer is listed in Table 2. §Monthly expenditure per person Low/Middle: <75000 JPY per month. Monthly expenditure High: =>75000 JPY per month. (100 JPY = $1 American dollar approx.).
